# Two Novel C-Terminus RUNX2 Mutations in Two Cleidocranial Dysplasia (CCD) Patients Impairing p53 Expression

**DOI:** 10.3390/ijms221910336

**Published:** 2021-09-25

**Authors:** Luca Dalle Carbonare, Franco Antoniazzi, Alberto Gandini, Silvia Orsi, Jessica Bertacco, Veronica Li Vigni, Arianna Minoia, Francesca Griggio, Massimiliano Perduca, Monica Mottes, Maria Teresa Valenti

**Affiliations:** 1 Department of Medicine, University of Verona, 37100 Verona, Italy; luca.dallecarbonare@univr.it (L.D.C.); silvia.orsi@univr.it (S.O.); jessica.bertacco@univr.it (J.B.); veronica.livigni@univr.it (V.L.V.); arianna.minoia@univr.it (A.M.); 2 Department of Surgery, Dentistry, Pediatrics and Gynecology, University of Verona, 37100 Verona, Italy; franco.antoniazzi@univr.it (F.A.); alberto.gandini@univr.it (A.G.); 3 Department of Neurosciences, Biomedicine and Movement Sciences, University of Verona, 37100 Verona, Italy; monica.mottes@univr.it; 4 Centro Piattaforme Tecnologiche, University of Verona, 37100 Verona, Italy; francesca.griggio@univr.it; 5 Biocrystallography Lab, Department of Biotechnology, University of Verona, 37134 Verona, Italy; massimiliano.perduca@univr.it

**Keywords:** cleidocranial dysplasia, bone, osteogenic differentiation, RUNX2

## Abstract

Cleidocranial dysplasia (CCD), a dominantly inherited skeletal disease, is characterized by a variable phenotype ranging from dental alterations to severe skeletal defects. Either de novo or inherited mutations in the *RUNX2* gene have been identified in most CCD patients. Transcription factor *RUNX2*, the osteogenic master gene, plays a central role in the commitment of mesenchymal stem cells to osteoblast lineage. With the aim to analyse the effects of *RUNX2* mutations in CCD patients, we investigated *RUNX2* gene expression and the osteogenic potential of two CCD patients’ cells. In addition, with the aim to better understand how *RUNX2* mutations interfere with osteogenic differentiation, we performed string analyses to identify proteins interacting with RUNX2 and analysed p53 expression levels. Our findings demonstrated for the first time that, in addition to the alteration of downstream gene expression, *RUNX2* mutations impair p53 expression affecting osteogenic maturation. In conclusion, the present work provides new insights into the role of *RUNX2* mutations in CCD patients and suggests that an in-depth analysis of the *RUNX2*-associated gene network may contribute to better understand the complex molecular and phenotypic alterations in mutant subjects.

## 1. Introduction

Cleidocranial dysplasia (CCD, OMIM119600) is a dominantly inherited skeletal disease characterized by complete penetrance with a variable phenotype. CCD is a rare disorder: its worldwide prevalence is: 1/1,000,000 and it can affect all ethnic groups [1]. The CCD related disorders vary from dental alterations to severe osseous diseases and osteoporosis [2]. Typical CCD-related features are the delayed closure of calvarial sutures as well as aplastic clavicles and dental anomalies. Patients may also show patent fontanelles, wormian bones, delayed pelvis ossification and short stature [1]. Mutations in *RUNX2* (Runt-related transcription factor 2) have been demonstrated to cause CCD [3]. *RUNX2* is the osteogenic master gene encoding the osteoblast-specific transcription factor and it plays a critical role in the commitment of mesenchymal stem cells to the osteoblast lineage [4]. Bone development fails in *RUNX2* knockout mice, despite normal cartilage development, and heterozygous RUNX2 mutant mice show CCD features, such as open fontanelles [5].

The *RUNX2* gene product is a 521 amino acid protein, characterized by functional domains, such as the glutamine/alanine-rich domain (QA), the Runt DNA domain (RHD), the nuclear localization signal (NLS), the proline/serine/threonine-rich domain (PST), the nuclear matrix targeting signal (NMTS), the repression domain (RD), and the VWRPY domain [6]. RUNX2 acts as a monomer or as a subunit of a heterodimeric complex with the core-binding factor β (CBFβ). CBFβ is also important in stabilization as its binding prevents ubiquitin-mediated degradation of RUNX2 [7]. Therefore, RUNX2 through the Runt domain binds to a conserved nucleotide sequence (R/TACCRCA) to induce the expression of specific osteogenic genes, such as COL1A1, SPP1, OCN, that code for collagen type 1, osteopontin and osteocalcin, respectively [8]. RUNX2 transcription factor is codified by an eight-exon gene, located on chromosome 6p21.1, controlled by two promoters: P1 and P2. Almost 40% of known mutations are missense and localized in the highly conserved Runt DNA-binding domain [9]. Mundlos et al. observed that insertion, deletion or missense mutations in *RUNX2* gene cause stop codons in the runt DNA-binding domain as well as in the C-terminal region [10]. *RUNX2* gene sequencing in CCD patients by Yoshida et al. demonstrated that missense mutations clustered mostly in the runt domain, with the exception of two *RUNX2* mutant CCD patients [11]. Genetic analyses performed in a group of Japanese patients identified 15 heterozygous mutations in 24 families and 1 polymorphism [11]. Most of the identified mutations were missense, and all mutations were located in the Runt-domain [11]. Extensive analysis in CCD patient groups identified several polymorphisms as well [11]. Recently, Papadopoulou et al. reported a case of hypophosphatasia in a child with a novel mutation in *RUNX2* and a pathogenic variant in the TNSALP gene [12]. Inherited or de novo genetic mutations in *RUNX2* have been identified in the majority of patients affected by CCD [13] [Other mutations in *RUNX2*, e.g., deletions, insertions, nonsense or splice-site variants observed along the entire gene, have been reported in CCD [14,15]. These mutations suggest that other domains, besides Runt, are important for osteogenesis. In fact, mutations in the RUNX2 carboxy-terminus induce alterations in SMAD interaction as well as in RUNX2 transcriptional activity [16]: mice lacking RUNX2 carboxy-terminus show a CCD-like phenotype [17]. RUNX2 transcriptional activity depends on nuclear localization and on subnuclear sites. The nuclear localization signal (NLS), composed of 9 amino acids (close to the Runt domain) and the nuclear matrix targeting signal (NMTS), composed of 31 amino acids (close to the protein carboxylterminus) direct the protein toward the nucleus or the nuclear matrix, respectively [18,19,20,21]. In particular, the localization of RUNX2 to subnuclear foci is fundamental for RUNX2-dependent transactivation [22]. Mutations in the NMTS domain disrupt RUNX2 subnuclear localization [23]: it has been demonstrated that mice lacking NMTS and carboxy-terminal residues are not capable of mineralization because the osteoblast maturation is prevented [17].

Moreover, basal RUNX2 expression is needed for correct proliferation and differentiation of osteoblasts. Many in vitro and in vivo studies as well as naturally occurring human genetic alterations, have demonstrated that perturbed RUNX2 expression causes serious consequences on the skeletal system [24].

With the aim to understand RUNX2 behavior in mutated CCD subjects, we analysed its expression in circulating progenitors of one of the two patients. Cultured cells of both were tested for *RUNX2* gene expression and for their osteogenic potential. In addition, by performing string analyses, we evaluated possible RUNX2 interactions with proteins regulating osteogenic maturation.

## 2. Results

### 2.1. Clinical Data

**Figure 1:**
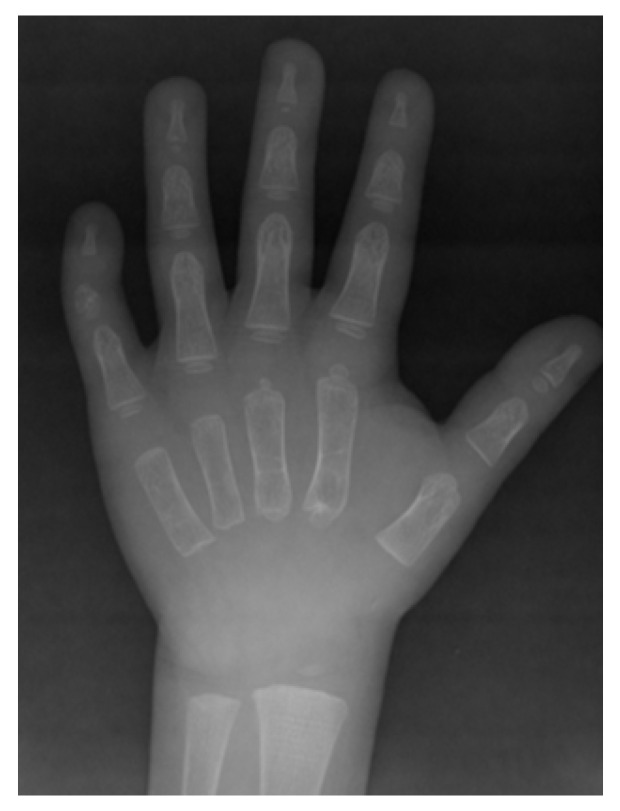
Radiologic image of right hand of patient 1. Note the absence of ossification of the carpal bones, in particular dysmorphism of the base of the second metacarpus and of the intermediate phalanx of the 5th finger.

Patient 1: On examination, the patient had a short stature (about −3/−4 SD) no axial deviations, flat feet, no scoliosis. Lower limb dysmetria (left < right of about 8 mm), prominent frontal drafts, late fontanel closure, typical facies, hypoplasia of the clavicles, delayed bone maturation (about 2 years) and absence of ossification of the carpal bones, in particular dysmorphism of the base of the second metacarpus and of the intermediate phalanx of the 5th finger (Figure 1). Increased bone turnover (CTX 0.805 ng/mL; n.v. (normal value) < 0.600 ng/mL) and hypovitaminosis D (25 ng/mL; n.v. > 30 ng/mL) were found.

**Figure 2:**
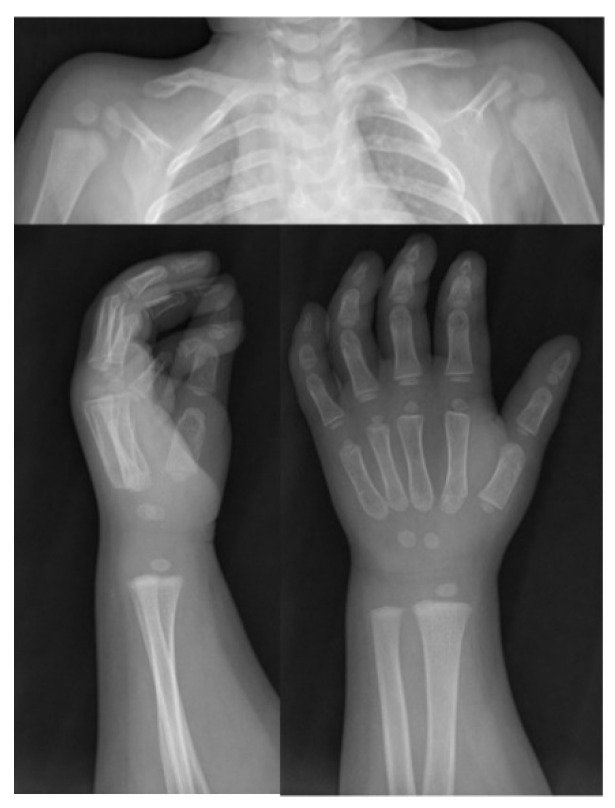
Radiologic images from patient 2. Note the mild hypoplasia of the right clavicle compared to contralateral, the delayed ossification in the carpus and dysmorphism at the level of the intermediate phalanx of the fifth finger and at the base of the second metacarpus.

Patient 2: On examination, delayed closure of the anterior fontanel and prominent frontal drafts, no axial deviations, no scoliosis, mild hypoplasia of the right clavicle, delayed ossification in the carpus, dysmorphism at the level of the intermediate phalanx of the fifth finger and at the base of the second metacarpus (Figure 2). Increased bone turnover (CTX 0.908 ng/mL; n.v. < 0.600 ng/mL) and hypovitaminosis D (19 ng/mL; n.v. > 30 ng/mL) were found.

### 2.2. RUNX2 Mutations

**Figure 3:**
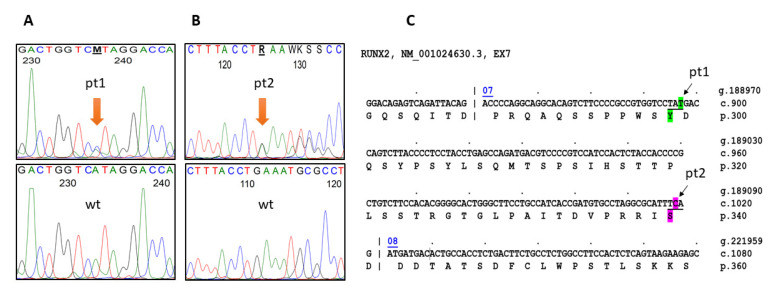
Reverse gDNA sequences of the two novel RUNX2 mutations in CCD patient 1 (**A**) and patient 2 (**B**) with their respective wild-type sequences. The mutations, a c.897T > G transition (Pt1) and a c.1019del of a C nucleotide (Pt2), highlighted in green and purple, respectively (**C**), were confirmed by forward gDNA sequencing.

In patient 1′s fibroblast DNA, we found the presence of a c.897T > G substitution leading to a premature stop-codon: p.(Tyr299*) in the c-terminus portion of RUNX2 protein (Figure 3A). Similarly, direct Sanger sequencing of patient 2′s DNA revealed the deletion of a C nucleotide in RUNX2 exon 7 (c.1019del), causing a reading frameshift and consequent premature stop codon: p.(Ser340*) (Figure 3B).

### 2.3. Modulation of RUNX2 and SPARC Expression in Pt2 cMSCs during Osteogenic Differentiation

**Figure 4:**
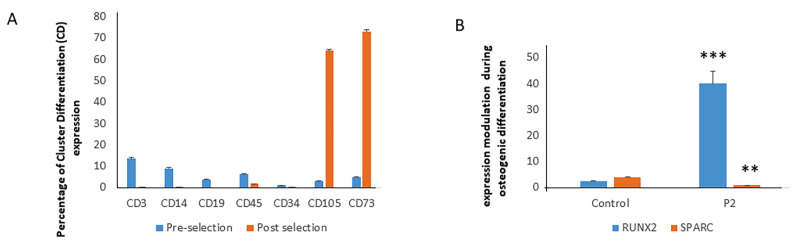
Mesenchymal Markers CD105 and CD73 were expressed to a percentage greater than 60% while hematopoietic markers were expressed at low levels (**A**). In cMSCs, following osteogenic differentiation, RUNX2 gene expression increased while SPARC decreased in Pt2 cMSCs compared to control (**B**). Two (for the evaluation of percentage of Cluster Differentiation expression) or three (for the evaluation of RUNX2 and SPARC expression) independent analyses in samples of each participant were performed in triplicate, and data are shown as mean ±standard deviation (SD). ** *p* < 0.005; *** *p* < 0.0001.

In patient 2, we were able to isolate cMSCs as demonstrated by the Cluster differentiation profile. In particular, the Mesenchymal Markers CD105 and CD73 were expressed to a percentage greater than 60% while the other Cluster Differentiation markers were either undetectable or expressed at low levels (Figure 4A). Then, we analysed RUNX2 and SPARC expression regulation during in vitro osteogenesis, comparing data with a control MSC line. In Pt2′s cMSCs, RUNX2 gene expression levels increased almost 40-fold, compared to a 2.3-fold increase in control after 7 days of osteogenic differentiation (*p* < 0.0001) (Figure 2). On the contrary, SPARC levels decreased in Pt2′s cMSCs compared to control after 7 days of differentiation (*p* < 0.005) (Figure 4B).

### 2.4. Osteogenically-Stimulated CCD Fibroblasts Express High Levels of RUNX2 Protein

**Figure 5:**
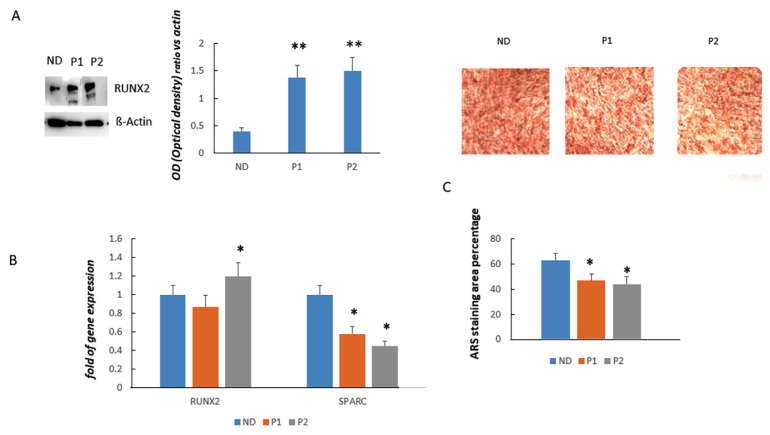
Western Blot shows RUNX2 protein levels in control (ND, normal donor), Pt1 (P1) and Pt2 (P2) os-fibroblasts (**A**). RUNX2 protein levels are higher in fibroblasts of CCD patients compared to control as shown by densitometric analyses (**A**). On the contrary, RUNX2 gene expression appears similar in os-fibroblasts of CCD patients and control. SPARC gene expression is lower in os-fibroblasts of CCD patients compared to control (**B**). Calcium deposition, evaluated by alizarin red staining (ARS), is reduced in os-fibroblasts of CCD patients compared to control (magnification: 10x) (**C**). All analyses were performed in samples of each participant in three independent analyses in triplicate. Data are shown as mean ± standard deviation (SD). * *p* < 0.05; ** *p* < 0.005.

In osteogenically-stimulated (os) fibroblasts from Pt1 and Pt2, we observed higher levels of RUNX2 protein compared to control (Figure 5A). However, RUNX2 gene expression was higher in Pt2 os-fibroblasts compared to control but not in Pt1 os-fibroblasts (Figure 5B). Despite the observed RUNX2 gene expression levels, SPARC gene expression was lower in CCD os-fibroblasts compared to control (Figure 3B). By performing alizarin red staining (ARS) to evaluate calcium deposition, we observed a reduced staining area in os-fibroblasts of CCD patients compared to control (Figure 5C).

### 2.5. P53 and PTEN Expression Are Reduced in CCD Os-Fibroblasts

**Figure 6:**
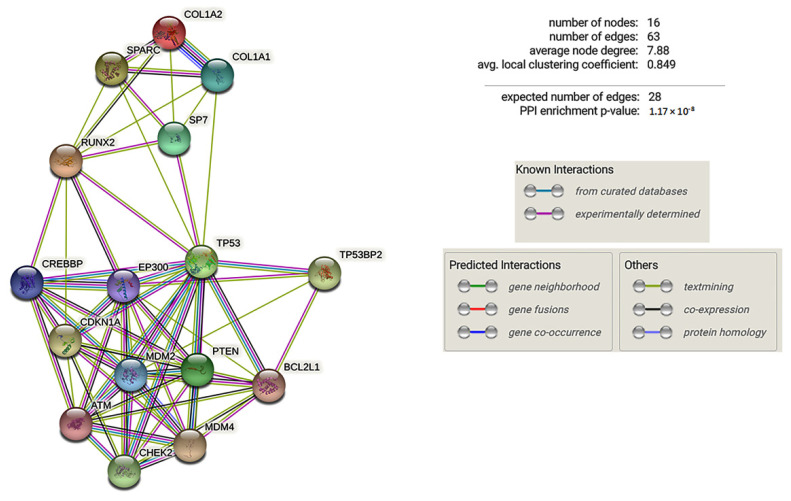
Functional protein interactions identified by the STRING portal. PPI enrichment *p*-value: 1.17 × 10^−8^.

To understand the effects of RUNX2 dysregulation in CCD patients’ cells, we investigated the expression levels of P53, which is known to play an important role in osteogenesis. First, we submitted RUNX2 and p53 gene products, as well as their respective regulated proteins, SPARC and PTEN, to the STRING portal (https://string-db.org/, assessed on 15 June 2021) for an independent inspection of potential connections between these proteins. Figure 6 shows the interaction between the functional proteins.

**Figure 7:**
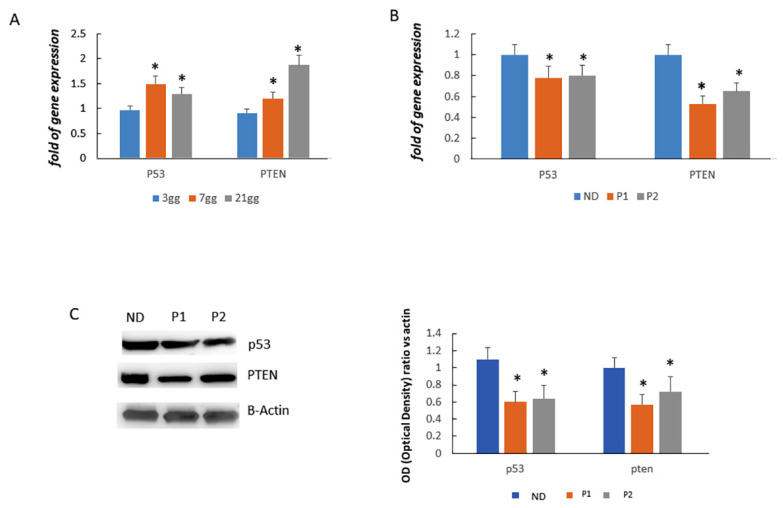
In a control MSC cell line, the expression of P53 and PTEN increased during osteogenic differentiation (**A**). p53 and PTEN gene expression was lower in CCD os-fibroblasts compared to control os-fibroblasts (ND = (normal donor) (**B**). Accordingly, both p53 and PTEN protein levels were lower in CCD os-fibroblasts compared to ND os-fibroblasts (**C**). All analyses were performed (in MSC line or in samples from each participant) in three independent analyses in triplicate. Data are shown as mean ±standard deviation (SD). * *p* < 0.05.

STRING analysis evidenced several biological processes among which the following functional gene ontology terms: Replicative senescence (GO:0090399), stress-induced premature senescence (GO:0090400), determination of adult lifespan (GO:0008340), and negative regulation of cell cycle arrest (GO:0071157). Therefore, we analysed p53 and PTEN expression in a control MSCs cell line and in CCD patients’ cells. As shown in Figure 7A, the expression of p53 and PTEN increased in the MSC cell line during osteogenic differentiation. In particular, we observed an increased expression of p53 and PTEN after 7 and 14 days, compared to 3 days of osteogenic differentiation. Instead, p53 expression was lower in CCD os-fibroblasts compared to os-fibroblasts of ND. Accordingly, the expression of PTEN, a gene regulated by p53, was lower in CCD os-fibroblasts compared to control os-fibroblasts (Figure 7B). p53 and PTEN protein levels were also lower in CCD os-fibroblasts compared to control os-fibroblasts (Figure 7C).

**Figure 8:**
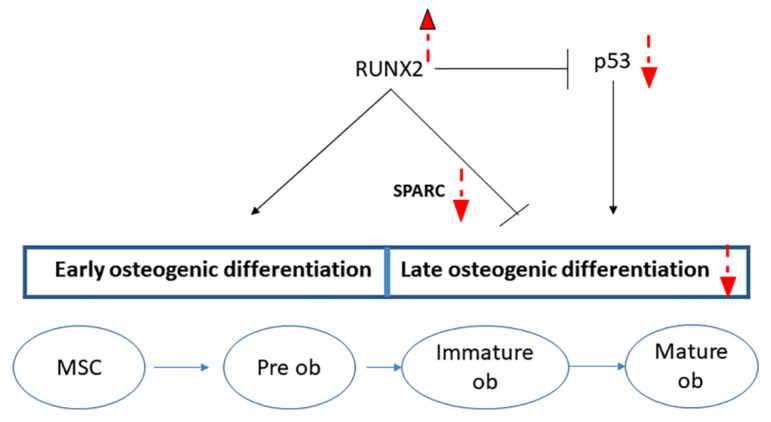
Schematic diagram showing (in red) the altered processes due to RUNX2 gene mutations in exon 7. Up (increased) and down (reduced) arrows showing the regulation in CCD patients.

Altogether, these data suggest that heterozygous RUNX2 mutations in both CCD patients impair osteogenic maturation by reducing the expression of RUNX2-downstream genes, such as SPARC, as well as by reducing p53 levels (Figure 8).

## 3. Discussion

Most RUNX2 mutations in CCD patients are localized in the runt domain: they are predominantly missense, preventing DNA binding, or nonsense generating truncated protein products. However, LOF (loss-of-function) mutations (e.g., nonsense and frame-shifts) have been identified throughout the gene [25]. Accordingly, in our study, we identified nonsense mutations in the c-terminus of RUNX2 in two CCD unrelated patients. A RUNX2 missense mutation has also been identified in exon 7 c.1259C→T[p.T420I] in a CCD Chinese family [26]. This threonine is conserved in vertebrates’ runt proteins; thus, its substitution suggests a pathogenic role [27]. The PST domain, spanning from exon 5 to exon 8, contains sequences for post-translational modifications; it is important for transactivation or transcription repression functions [27]. Therefore, mutations in the PST domain may possibly impair transactivation or the transcription repression function of RUNX2.

In particular, we identified two novel c.897T> G-> p.(Tyr299*) and c.1019del-> p.(Ser340*) LOF mutations. In the first case, the mutation causes the substitution of Tyrosine 299 with a STOP codon, and in the second case, the mutation causes a C deletion resulting in a frameshift creating a premature STOP codon. Both mutations cause the loss of PST domain (ranging from AA 303-596) in heterozygosis. The PST domain contains several sequences important for post-translational regulation by the extracellular signal-regulated kinase 1 and 2 (Erk1/2), the protein kinase A (PKA), and the Akt kinases [6]. In addition, NMTS, localized inside of PST domain, regulates the association of RUNX2 to subnuclear locations in the nuclear matrix and it is important for RUNX2 activity [6]. It has been demonstrated that mice lacking NMTS and the remaining C-terminus are unable to produce bone as the osteoblastic maturation is prevented, suggesting that the NMTS region is important for RUNX2 function in vivo [28]. Other important sequences cut out by the reported mutations are AD3 (a transactivation domain) and RD (repression domain), both contained in PST sequence [29].

Despite the observed overexpression of RUNX2 gene in Pt.2 cMSCs as well as increased RUNX2 protein levels in both CCD patients os-fibroblasts, we found reduced RUNX2 transcriptional activity in their cultured cells. Other authors, observed that mutations targeting RUNX2 carboxy-terminus impair RUNX2 transcriptional activity [16]. Accordingly, we observed that the expression of SPARC, a target gene of RUNX2, was reduced in cells of CDD patients. SPARC gene product, osteonectin, is a secreted acidic protein rich in cysteine, one of the most abundant non-collagenous proteins of mineralized tissues [30]. It has been demonstrated that SPARC-null mice are affected by intervertebral disc degeneration and that they are nonresponders to bone-anabolic parathyroid treatment [31,32]. Reduced mineralization in cells with reduced osteonectin levels is expected. In fact, reduced SPARC gene expression was associated with a reduced calcification, evaluated by Alizarin red staining, in os-cells of CDD patients. These findings might justify their skeletal features, such as clavicle hypoplasia, delayed bone maturation or absence of ossification of the carpal bones, as well as skeletal dysmorphisms. The observed increased levels of RUNX2 protein are in contrast with ascertained RUNX2 haploinsufficiency in CCD patients. However, haploinsufficiency may produce different complex scenarios [33]. If mutations cause the carboxyl-end removal, the resulting truncated protein can act as a self-defeating polypeptide, while nonsense mutations can produce an inactive peptide [33]. Only mutations preventing transcription or mRNA expression or encoding unstable fragments suggest that haploinsufficiency is the disease-causing mechanism. Our data instead demonstrate that RUNX2 mutations in exon 7 may lead to protein overproduction. A limitation of our study might be the lack of a female paediatric control. However, we have previously reported that RUNX2 expression is associated with age-related changes in bone mineral density in the young population (age 15–50), and we have observed gender differences only starting from age 20 [34]. In addition, Glastre et al., by analysing Bone Mineral Content in 135 healthy children aged 1–15 years, reported that no significant differences between boys and girls were observed until age 12 for BMD [35]. Therefore, in healthy prepubertal individuals, gender-associated RUNX2 expression differences are not expected, and the 10-year-old child that we recruited for the study can be considered suitable for comparing the data with the two CCD paediatric patients. Hence, the observed increased levels of RUNX2 protein in os-fibroblasts of both CCD patients might be the consequence of compensation mechanisms for the reduced expression of downstream osteogenic genes, such as SPARC, or of the accumulation of dysfunctional RUNX2. Notably, overexpression of RUNX2 can compromise bone quality, as we have demonstrated in acromegaly patients [36,37]. The double-edged sword role of p53 in osteogenesis has been demonstrated already. In fact, p53 affects osteogenic commitment and promotes terminal osteogenic differentiation [38]. Recently, Zhou et al., demonstrated that Wnt/ß-catenin-mediated p53 suppression is indispensable for osteogenic differentiation of mesenchymal progenitor cells [39]. Therefore, with the aim to investigate RUNX2 mutations effects on p53 expression, we first explored putative protein interactions by using the STRING portal. Our bioinformatics data highlighted a strong interaction between RUNX2 and p53 and identified several biological processes regulated by their interplay, such as replicative senescence, stress-induced premature senescence, determination of adult lifespan and negative regulation of cell cycle arrest among others. By using the CRISPR/Cas9 technology, we have previously observed that in Runt KO melanoma cells p53 expression and protein levels are increased compared to WT cells, demonstrating that RUNX2 is a negative regulator of p53 [40]. Ozaki et al. suggested that RUNX2 may reduce p53 transcriptional activity by acting with HDAC6 [41]. In addition, it has been demonstrated that PTEN is a downstream p53 gene [42]. Accordingly, by performing in vitro experiments with an MSC cell line, we observed an increased expression of p53 and PTEN genes during osteogenic differentiation. By contrast, we observed lower expression levels of both p53 and PTEN mRNAs and proteins, respectively, in CCD cells compared to control. These data are in agreement with the reduced calcification in CCD cells demonstrated by Alizarin red staining, suggesting that RUNX2 dysregulation prevents osteogenic maturation by reducing p53 levels too. We believe that these are important aspects to consider for a thorough understanding of skeletal alteration pathogenesis in CCD patients.

## 4. Material and Methods

### 4.1. Patients

Two pre-pubertal patients, a, 8-year-old male and a 10-year-old female clinically diagnosed with CCD, were examined. Peripheral blood and skin biopsies were collected from CDD patients and from an age matched healthy donor (male, 10 years old). Informed consent was obtained from parents of each patient and donor, and children gave their assent for care. The study was approved by the ethical committee of Azienda Ospedaliera Universitaria Integrata of Verona, Italy (number 1538; 3 December 2012; local ethical committee of Azienda Ospedaliera Integrata di Verona).

### 4.2. Circulating Mesenchymal Stem Cells (cMSCs)

cMSCs were obtained from peripheral blood as previously reported by us [36]. In particular, cMSCs were isolated from 50 mL of peripheral blood using a method with two Ficoll procedures to deplete hematopoietic cells by antibodies cocktail. Firstly, mononuclear cells were obtained from heparinizated peripheral blood by centrifugation. Then, concentrated mononuclear cells were mixed with 4 mL of additional peripheral blood (from the same patient) to apply 200 μL of an antibodies cocktail (RosetteSep Mesenchymal Enrichment Cocktail; code #15128, StemCells, Vancouver, BC, Canada); this RosetteSep Mesenchymal Enrichment Cocktail contains bi-specific antibody against red blood cells (glycophorin A) and CD3-, CD14-, CD19-, CD38- and CD66b-positive cells (Stem Cell Technologies, Inc., Vancouver, BC, Canada). These antibodies crosslinked the unwanted cells with red blood cells, causing them to pellet together when centrifuged over the second Ficoll.

cMSCs were cultured at first in MesenPRO RSBasal Medium (Gibco, Thermo Fisher Scientific, Waltham, MA, USA) additioned with MesenPRO RSGrowth Supplement (Gibco) and then it was induced the differentiation for 7 days towards the osteogenic lineage using StemProOsteocyte Differentiation Basal Medium (Gibco) additioned with 1% PSA (Penicillin-Streptomycin-Amphotericin, Thermo Fisher Scientific, Waltham, MA, USA).

### 4.3. Analysis of Cell Phenotype

To compare pre- and post-depleted samples, we analysed the expression of CD3, CD14, CD19, CD45, and CD34 hematopoietic markers at RNA levels as previously reported [22]. We also analysed MSC-positive markers CD73 and CD105. This method allows the phenotypic analysis of cells obtained with stringent stem cell purification techniques [43].

### 4.4. Fibroblasts

Fibroblast cells were isolated from skin biopsy taken from the patients and a healthy donor, immediately rinsed with 1× PBS containing 1% antibiotics (Penicillin-Streptomycin-Amphotericin), cut into small pieces and plated in culture flasks containing 1× DMEM (Dulbecco’s Modified Eagle’s Medium) complete medium (20% FBS (fetal bovine serum) + 1% Glutamax + 1% antibiotic) under sterile conditions. After 2 weeks, fibroblasts grown out of the tissues were collected, plated in new culture flasks containing 1× DMEM complete medium with 10% FBS and allowed to grow until they reached 80% confluency, adding fresh new medium every other day.

Finally, cells were treated with 10 mM of β-glycerophosphate and 50 μgr/mL of ascorbic acid (Sigma St. Louis, MO, USA) for 7 days as previously described [44]. Then, cells were collected, and the pellet was stored until use.

### 4.5. DNA Extraction

We performed DNA extraction from dermal fibroblasts and MSC dry pellets using the QIAampDNA Blood Mini Kit (Qiagen, Milan, Italy) following the manufacturer’s protocol.

DNA quantification was done using Qubit 4 Fluorometer (Invitrogen, Thermo Fisher Scientific, Waltham, MA, USA) with QubitdsDNA HS Assay Kit (Invitrogen, Thermo Fisher Scientific, Waltham, MA, USA).

### 4.6. Sanger Sequencing

We confirmed the presence of the two c-terminus RUNX2 mutations, initially identified in the CCD patients by “Casa del sollievo e della sofferenza” U.O.C. Genetica Medica—S. Giovanni Rotondo (FG)-Italy, by targeted sanger sequencing of a 470 base-pair (bp) PCR product corresponding to exon 7 and adjacent intron regions of RUNX2 gene (NM_001024630.3). Specific primers (purchased from Thermo Fisher Scientific, Waltham, MA, USA) were designed using Primer3 and BLAST (NCBI, https:www.ncbi.nlm.nih.gov/tools/primer-blast/, accessed on 3 February 2019) and were the following: Forward 5′-TAAGGCCTGAAAGGATGGGGT-3′ and Reverse 5′-ATGTGGGCAAGGGAATGACAA-3′. PCR was conducted with Mastercycler^®^ ep Gradient S^®^ (Eppendorf, Milan, Italy) and GoTaq^®^ Hot Start Polymerase kit (Promega, Madison, WI, USA) by 2 min at 96 °C followed by 35 cycles of 96 °C for 30 s, 60 °C for 30 s and 72 °C for 30 s. A final step of 72 °C for 5 min concluded the PCR program. Purity and identity (by means of length in bp) of PCR product was checked by 1.5% agarose and gel electrophoresis. After purification with FastGene™ kit (Nippon Genetics, Mariaweilerstraße, Düren, Germania), 1.5 µL of the PCR product was used with Dye Terminator Cycle Sequencing (DTCS), Beckman Coulter; Fullerton, CA, USA) Quick Start Kit (Sciex, Milan, Italy) following the manufacturer’s instructions.

### 4.7. RNA Extraction

We performed RNA extraction from dermal fibroblasts and MSC dry pellets using the RNeasyProtect Mini Kit (Qiagen, Milan, Italy) following the manufacturer’s protocol. Three hundred fifty microliters of Buffer RLT and 350 μL of ethanol 70% were added to the dry pellets. The solution was transferred into an RNeasy Mini spin column placed into a 2 mL collection tube and spun for 15 s at 8000× *g*, and the flow-through was subsequently discarded. Then, we added 700 μL of Buffer RW1 to the RNeasy spin column, centrifuged for 15 s at 8000× *g* and discarded the flow-through. For two times, we added 500 μL of Buffer RPE and centrifuged at 8000× *g*, spinning for 15 s the first time and 2 min the second time. To dry the membrane, we spun at full speed for 1 min. For elution 50 μL of RNase-free water were added directly to the membrane and centrifuged for 1 min at 8000× *g*. RNA quantification was obtained using a Qubit 4 Fluorometer (Invitrogen, Thermo Fisher Scientific, Waltham, MA, USA) with QubitRNA HS Assay Kit (Invitrogen, Thermo Fisher Scientific, Waltham, MA, USA).

### 4.8. Reverse Transcription and Real Time PCR

We used the High Capacity cDNA Reverse Transcription Kit (Applied Biosystems, Waltham, MA, USA) following the manufacturer’s instructions as previously described [45]. Gene expression evaluation in fibroblasts was performed using real-time PCR. The Real-Time PCR was performed, following the manufacturer’s protocol, using the TaqMan Universal PCR Master Mix (Thermo Fisher Scientific, Waltham, MA, USA) and the following commercially pre-designed probes (MIQE guidelines compliant, Thermo Fisher Scientific, Waltham, MA, USA) (Thermo Fisher Scientific, Waltham, MA, USA, code#4331182): (RUNX2,Hs00231692_m1; SPARC, Hs00234160_m1; CD3, Hs00174158_m1; CD14, Hs02621496-s1; CD19, Hs00174333_m1; CD45, Hs00174541_m1; CD34, HS00156373_m1; CD73, Hs00159686_m1, CD105, Hs00923996_m1; TP53, Hs01034249_m1; PTEN, hs02621230m1; ACTB, 4326315E; B2M, 4326319E; Applied Biosystems). Rather than a single gene, we used ACT-β and β2M to ensure an accurate normalization of gene expression analyses as suggested by Vandesompele et al. [46]. The amplification was performed using QuantStudio3 Real Time PCR System (Thermo Fisher Scientific) as previously reported [45]. The PCR stage was repeated for 40 cycles. The data were analyzed by QuantStudio Design & Analysis software (VERSION 1.4.3, Life Technologies Corporation, Carlsbad, CA, USA). In particular, at least three independents analyses were performed, and each analysis was performed in triplicate with Ct values averaged. Because Ct values vary linearly with the logarithm of the amount of RNA, this average represents a geometric mean.

### 4.9. Digital Droplet PCR

In order to analyse the expression of RUNX2 and SPARC in cMSCs, we performed a digital droplet PCR (ddPCR) test on Pt2 and Control. Five microliters of cDNA (0.2 ng/µL) were added to 10 µL of ddPCR supermix for no UTP probes, and to 1 µL of RUNX2 (FAM-NGB) and B2M (VIC-NGB) TaqMan probe (Applied Biosystems, Waltham, MA, USA). The mix was applied to the QX200 droplet generator (BioRad, Hercules, CA, USA) with 70 µL of oil. Droplets were transferred into a 96-well plate and heat-sealed with a tinfoil sheet.

Thermocycling conditions were as follows: Step 1: pre-incubation at 95 °C for 10 min, Step 2: amplification at 95 °C for 30 s, Step 3: annealing at 60 °C for 1 min, Step 4: heat inactivation at 98 °C for 10 min; Step 2 and 3 were repeated for 40 cycles. Plates containing droplets were placed in a QX200 droplet reader, which analyses droplets individually, through a two-color detection system (FAM and VIC, Hercules, CA, USA). Results were processed by QuantaSoft (1.7.4.0917 © 2021, Bio-Rad, Hercules, CA, USA) according to the manufacturer’s instructions.

### 4.10. Protein Extraction and Western Blot

The dry pellet, which was obtained by washing cells with PBS by centrifugation at 4000× *g* for 5 min, was suspended in cold Pierce RIPA Buffer 1X (Thermo Fisher Scientific) additioned with protease inhibitor cocktail tablets (cOmplete) and kept in ice for 40 min. Proteins were obtained by collecting the supernatant after sample centrifugation at 10,000× *g* for 40 min at 4 °C. Protein concentration was determined using a BCA Protein Assay Kit (Thermo Fisher Scientific, Waltham, MA, USA). Western Blot procedures were performed as previously reported [45]. In brief, protein samples were diluted in 4× Laemmli’s sample buffer (Bio-Rad, Hercules, CA, USA) and heated for 5 min at 95 °C. Then, proteins were separated by sodium dodecyl sulfate–polyacrylamide gel electrophoresis (SDS–PAGE), using a mini-PROTEAN^®^ TGX™ Precast gradient 4–20% gel (Bio-Rad, CA, USA). Proteins were transferred onto polyvinylidene difluoride (PVDF) membranes (Thermo Fisher Scientific, Waltham, MA, USA). PVDF membranes were probed with the primary and secondary following antibodies: β-Actin, 1:1000 Actin (mAbGEa) mouse mAb (code:#MA1-744, Invitrogen); RUNX2, 1:1000 RUNX2 (D1H7) rabbit mAb (code:#8486, Cell Signaling Technology, Danvers, MA, USA), p53, 1:1000 p53 (1C12) mouse mAb (code:#2524, Cell Signaling), PTEN, 1:1000 mAb rabbit (code:#138G6, Cell Signaling Technology); Anti Mouse,1:1000 Anti-mouse IgG HRP-linked Antibody (code:#7076S, Cell Signaling Technology); Anti Rabbit, 1:1000 Anti-rabbit IgG HRP-linked Antibody (code:#7074S, Cell Signaling Technology). To reveal the chemiluminescence signal, we used ECL Select Western Blotting Detection Reagent (GE, Buckinghamshire, UK) using the Alliance Q9 System (UVITEC, Cambridge, UK) with Nine Alliance software (UVITEC, Cambridge, UK). Densitometric analyses were performed using ImageQuant software (GE Healthcare, Little Chalfont, UK), and the relative protein band intensity was normalized to β-actin [36].

### 4.11. Alizarin Red Staining

We performed Alizarin red staining as previously described [47]. Briefly, after 14 days’ culture under osteogenic stimulation, fibroblasts were fixed with 70% ethanol and, after washing, cells were stained with 40 mM of Alizarin red S for 5 min at pH 4.1 and rinsed for 15  min with 1x phosphate-buffered saline. The stained area was analysed by using ImageJ software (NIH, Bethesda, MD, USA) as previously reported [47].

### 4.12. Statistical Analysis

Results were expressed as mean ± SD. Statistical analysis was assessed by Student’s paired-test. Differences were considered significant with *p* < 0.05. Analyses were carried out in two or three independents analyses, performed in samples from each participant in triplicate. Statistical analyses were performed using SPSS for Windows, version 22.0 (SPSS Inc., Chicago, IL, USA).

## 5. Conclusions

In conclusion, our data demonstrated that the two c-terminus nonsense mutations in RUNX2 never described before affect osteogenic differentiation. The impaired osteogenic differentiation is in part a consequence of the reduced expression of RUNX2- downstream genes. However, for the first time we highlighted the involvement of p53 dysregulation in CCD disease. In fact, our study suggests that RUNX2 mutations affect osteogenesis not only by dampening the expression of genes coding for skeletal matrix proteins, but also by tuning down p53, thus preventing osteogenic maturation. Therefore, the awareness that RUNX2 mutations may impair signaling pathways opens new perspectives for therapeutic targets, and it may have important implications for CCD clinical treatment.

## Data Availability

The datasets used and/or analyzed during the current study are available from the corresponding author upon a reasonable request.
